# The Role of Mitochondrial Dysfunction and Dynamics in Hypertensive Heart Disease: Mechanisms and Recent Advances

**DOI:** 10.3390/biology14091212

**Published:** 2025-09-08

**Authors:** Bislom C. Mweene, Hanzooma Hatwiko, Joreen P. Povia, Sepiso K. Masenga

**Affiliations:** 1 Department of Cardiovascular Science and Metabolic Diseases, Livingstone Center for Prevention and Translational Science, Livingstone 10101, Zambia; bischarm1st@gmail.com (B.C.M.); hhatwiiko@mu.edu.zm (H.H.); 2 Department of Health Economics, Livingstone Center for Prevention and Translational Science, Livingstone 10101, Zambia; joreenpovia@lcpts.org; 3 Department of Molecular Physiology & Biophysics, Vanderbilt University, Nashville, TN 37232-0615, USA

**Keywords:** left ventricular hypertrophy, mitochondrial dysfunction, aging, oxidative stress, mitophagy, metabolic reprogramming, hypertensive heart disease, fusion, fission

## Abstract

Hypertensive heart disease (HHD) is characterized by pressure overload-induced cardiac remodeling, in which mitochondrial dysfunction has emerged as a central contributor to pathophysiology. Mitochondria occupy roughly one-third of the volume of a cardiomyocyte and serve as the primary source of ATP for the constantly active heart, while also regulating calcium homeostasis, redox balance, and apoptotic signaling. Chronic hypertension imposes energetic and oxidative stress on cardiomyocytes, disrupting mitochondrial structure and function. Key mitochondrial quality control processes including organelle fusion–fission dynamics, biogenesis, and mitophagy become dysregulated in HHD, leading to impaired energy production and heightened cell injury. This unstructured review discusses the physiological roles of mitochondria in cardiac muscle and examines how altered mitochondrial dynamics contribute to hypertensive cardiac damage. We detail mechanisms of mitochondrial dysfunction in HHD, such as excessive fission, cristae disruption, and oxidative stress, and how these changes are exacerbated by aging. Age-related mitochondrial remodeling such as loss of cristae and decreased organelle volume may synergistically worsen hypertensive cardiac injury. We further integrate findings from recent studies in animal and human models, including advanced three-dimensional ultrastructural analyses and molecular investigations that illuminate new aspects of mitochondrial network organization, the mitochondrial contact site and cristae organizing system (MICOS), cristae maintenance complex, and quality control pathways in HHD. Understanding mitochondrial dysfunction in HHD reveals potential therapeutic avenues targeting mitochondrial quality and dynamics to preserve cardiac function in hypertension.

## Simple Summary

Chronic high blood pressure puts dangerous stress on the heart, leading to a condition called hypertensive heart disease. A key problem is damage to the tiny power plants inside heart muscle cells, called mitochondria, which normally provide energy and help the heart work properly. This review aimed to understand how these essential power plants become faulty under high blood pressure and how this damage contributes to heart injury. The findings show that high blood pressure disrupts vital processes that keep mitochondria healthy, causing them to break apart incorrectly, lose their internal structure, produce harmful substances, and fail to make enough energy. These problems get significantly worse as people get older. The conclusion is that this mitochondrial damage is one of the major reasons why high blood pressure harms the heart. Understanding exactly how these power plants fail is valuable because it points scientists towards developing new treatments focused on protecting or repairing mitochondria. Such treatments could ultimately help millions of people with high blood pressure avoid serious heart damage and maintain a healthier heart for longer.

## 1. Introduction

Hypertensive heart disease, defined by the structural and functional cardiac changes induced by chronic high blood pressure, remains a leading cause of heart failure and mortality worldwide [1]. Persistent hypertension drives left ventricular hypertrophy, fibrosis, and eventually ventricular dysfunction. A growing body of evidence implicates mitochondrial dysregulation as a pivotal mediator linking the hemodynamic stress of hypertension to maladaptive cardiac remodeling [1,2]. Mitochondria are not only the “powerhouses” that supply ATP to meet myocardial energy demands, but also dynamic regulators of reactive oxygen species (ROS) generation, calcium signaling, and cell death pathways [3]. In the hypertensive heart, these organelles undergo structural and functional alterations: studies have documented decreased mitochondrial density and mass, swollen and fragmented mitochondria with cristae loss, and respiratory dysfunction in animal models of hypertension [1,4,5]. Such mitochondrial injury can create an energy deficit and trigger oxidative stress and apoptotic signaling, contributing to cardiomyocyte dysfunction and loss [1].

Mitochondrial health is maintained by a balance of biogenesis, fission and fusion dynamics, and selective autophagy (mitophagy). Hypertensive stress can upset this balance; for instance, excessive mitochondrial fission and inadequate mitochondrial renewal have been observed in pressure-overload cardiac hypertrophy models [3,6,7]. Moreover, the risk of HHD increases with age, as aging itself is associated with a decline in mitochondrial function and quality control [8,9]. The aging heart accumulates mitochondrial DNA damage and oxidative injuries and shows altered mitochondrial dynamics, which may lower the threshold for hypertensive damage [10]. Thus, in an elderly hypertensive patient, mitochondrial dysfunction from both chronic pressure overload and aging can synergistically precipitate heart failure.

This review provides a comprehensive overview of the role of mitochondrial dysfunction and dynamics in hypertensive heart disease. We first outline the physiological roles of mitochondria in cardiac muscle and the mechanisms of mitochondrial dynamics (fission, fusion, biogenesis, and mitophagy) that preserve cardiomyocyte health. We then examine how hypertension deranges these processes, detailing the structural mitochondrial alterations, bioenergetic deficits, and signaling abnormalities observed in HHD. The influence of aging on cardiac mitochondria is discussed to highlight the intersection of age-related mitochondrial changes with hypertensive cardiopathy. Finally, we summarize recent advances from both animal models and human studies that shed new light on mitochondrial biology in hypertensive heart disease, including novel insights into mitochondrial network regulation [11], cristae organization complexes [12], and cutting-edge imaging of mitochondrial ultrastructure in failing human hearts [13]. By integrating these findings, we hope to illuminate emerging mechanisms and therapeutic targets aimed at mitochondria for improving outcomes in hypertensive heart disease.

## 2. Methods

This narrative review was conducted by systematically searching the PubMed, Scopus, and Web of Science electronic databases for relevant English-language articles published up to 2025. The search strategy utilized a combination of the following key terms and Medical Subject Headings (MeSH): “hypertensive heart disease”, “hypertensive cardiac hypertrophy”, “heart failure”, “mitochondrial dysfunction”, “mitochondrial dynamics”, “mitochondrial fission”, “mitochondrial fusion”, “mitophagy”, “mitochondrial biogenesis”, “oxidative stress”, “reactive oxygen species”, “aging”, “mitochondria”, and “cardiomyopathy.” The reference lists of retrieved articles were also screened for additional relevant publications. Articles were selected based on their relevance to the focus of this review, specifically, the role of mitochondrial biology in the pathophysiology of hypertensive heart disease. Priority was given to original research articles, both preclinical and clinical, as well as authoritative reviews and meta-analyses.

## 3. Physiological Roles of Mitochondria in Cardiac Muscle

Mitochondria are indispensable for normal cardiac physiology. In adult cardiomyocytes, mitochondria constitute roughly 30% of cell volume, reflecting the massive energetic demands of continuous rhythmic contraction [14]. These organelles are organized in a dense network between myofibrils and beneath the sarcolemmal membrane to efficiently deliver ATP to sites of actin–myosin ATPase activity. The primary role of cardiac mitochondria is to generate ATP through oxidative phosphorylation. Fatty acids are the dominant fuel under resting conditions, with glucose and lactate contributing as additional substrates. Mitochondrial oxidative metabolism must rapidly adjust to changes in workload, from basal metabolism to exercise-induced tachycardia, to sustain contractile function [15].

Beyond ATP production, mitochondria fulfill several other critical roles in cardiomyocytes. They help buffer cytosolic calcium, especially during excitation–contraction coupling, by taking up Ca^2+^ when cytosolic levels are high and releasing it slowly, thus modulating calcium transients and preventing cytotoxic Ca^2+^ overload [16,17,18]. Mitochondria also participate in the synthesis and breakdown of key metabolites (e.g., citrate, glutamate, lipid intermediates) and are integrally involved in redox homeostasis through the generation and scavenging of ROS [19,20,21]. Controlled ROS signaling from mitochondria can influence cellular processes, whereas excessive ROS can trigger oxidative damage. In addition, mitochondria house the apoptotic pathway apparatus; release of pro-apoptotic factors like cytochrome c from mitochondria initiates cardiomyocyte apoptosis in response to irreparable stress [22,23].

The structure and connectivity of the mitochondrial network in cardiac muscle are specialized for these roles. Mitochondria in cardiomyocytes form a reticular grid confined by the myofibrillar architecture, which maximizes ATP diffusion distances of only a few microns [24]. Recent work even suggests that muscle cells can exhibit distinct mitochondrial network configurations governed by specific genetic factors [11]. For example, we have previously shown that evolutionarily conserved transcriptional regulators (e.g., cut, salmon/salm, and H15 in Drosophila) determine whether muscle mitochondria form parallel arrays or more branched, grid-like networks, independent of muscle fiber type [11]. Intriguingly, these regulators have mammalian homologs such as Cut Like Homeobox 1 (CUX1), Spalt Like Transcription Factor 1 and 2 (SALL1/2), and various Homeobox (HOX) genes, suggesting the existence of a conserved genetic framework for orchestrating mitochondrial network topology. While this study was conducted in Drosophila flight muscle, it raises the compelling possibility that analogous transcriptional programs are active in mammalian hearts to maintain the precise alignment of mitochondria along sarcomeres. Dysregulation of such programs by hypertrophic signaling pathways in hypertension could be a previously overlooked mechanism contributing to mitochondrial network disorganization [25,26].

Crucially, mitochondrial function in the heart is tightly linked to mitochondrial morphology and ultrastructure. The inner mitochondrial membrane is folded into cristae, where respiratory complexes reside. The cristae architecture provides a large surface area for electron transport and ATP synthase activity [27]. In cardiac muscle, cristae density is normally high, supporting robust ATP output [24]. Structural components like the MICOS complex and the inner membrane fusion protein optic atrophy 1 (OPA1) preserve cristae integrity and optimize mitochondrial efficiency [28,29]. In summary, healthy cardiac mitochondria are abundant, structurally well-organized, and dynamically responsive organelles that supply energy and maintain cellular homeostasis. The heart’s reliance on proper mitochondrial function means that even subtle mitochondrial defects can significantly impact cardiac performance, an effect magnified in the face of stressors like hypertension.

Furthermore, emerging evidence suggests that mitochondrial functional specialization exists within the same cell. A recent paradigm-shifting study by Ryu et al. (2024) demonstrated that within cardiomyocytes, distinct mitochondrial subpopulations can be dedicated to specific metabolic tasks, with some primarily generating ATP while others are specialized for the biosynthesis of molecules like ornithine and proline [30]. This metabolic heterogeneity, governed by local cellular ATP demand, adds a new layer of complexity to our understanding of mitochondrial biology in the heart. In the context of HHD, it is plausible that pressure overload and metabolic stress could disrupt this sophisticated functional partitioning, contributing to the overall bioenergetic crisis. Future research investigating the fate of these specialized subpopulations in hypertension could reveal novel disease mechanisms and therapeutic targets.

## 4. Mitochondrial Dynamics: Fission, Fusion, Biogenesis, and Mitophagy

Mitochondria are not static entities, they continually undergo dynamic processes of fission and fusion, as well as turnover via biogenesis and mitophagy. These mitochondrial dynamics are essential for the quality control and adaptability of the mitochondrial network [1]. In the heart, a finely tuned balance of fission and fusion helps meet changing energetic demands and remove damaged mitochondrial components.

### 4.1. Fusion

Mitochondrial fusion is the merging of two mitochondria into one, allowing mixing of contents (mtDNA, proteins, metabolites) between organelles. This process helps buffer stress by diluting damaged components and sharing metabolic intermediates. Mitochondrial fusion in mammals is mediated by large GTPases: mitofusin 1 and 2 (Mfn1/2) on the outer membrane, which tether two mitochondria together, and OPA1 on the inner membrane, which mediates inner membrane fusion and cristae remodeling [31,32,33]. In cardiomyocytes, Mfn2 also facilitates physical tethering between mitochondria and the endoplasmic reticulum, important for calcium signaling [34]. Proper fusion is required to maintain elongated, interconnected mitochondria capable of optimal oxidative phosphorylation [35]. If fusion is impaired (for example, by loss of Mfn2 or OPA1), mitochondria become overly fragmented, and cardiomyocytes can exhibit energy deficiency and heightened apoptosis susceptibility [36,37].

### 4.2. Fission

Mitochondrial fission is the division of one mitochondrion into two progenies. Fission serves to create new organelles (especially in dividing cells) and to isolate damaged regions of mitochondria that can then be removed by autophagy. The master regulator of fission is the dynamin-related protein 1 (Drp1), a cytosolic GTPase that is recruited to mitochondrial outer membrane sites marked by receptors such as Fis1, MFF, and MiD49/51 [38]. Drp1 forms helices that constrict and sever the mitochondria. In the heart, excessive activation of Drp1 has been linked to pathological fragmentation of mitochondria. Notably, fission is a double-edged sword: while moderate fission maintains healthy mitochondrial numbers and facilitates the removal of defective parts, excessive fission can lead to small, bioenergetically compromised mitochondria and can trigger apoptosis [39,40]. Mitochondrial dynamics regulate key cellular processes, including ATP generation, ROS production, calcium homeostasis, and apoptosis, underscoring why an imbalance in fission/fusion is detrimental.

### 4.3. Biogenesis

Mitochondrial biogenesis is the process by which new mitochondria are formed, involving the growth and division of pre-existing mitochondria and the import of nuclear-encoded proteins. This process is orchestrated by transcriptional coactivator PGC-1α along with transcription factors such as NRF1/2 and estrogen-related receptors that induce the expression of mitochondrial proteins and of mitochondrial DNA replication factors like TFAM [41,42]. In the heart, biogenesis is upregulated during increased workload (exercise, thyroid hormone stimulation) to boost the organelle content and meet energy demands. Conversely, reduced biogenesis contributes to mitochondrial insufficiency in disease. For instance, hypertrophied hearts and aged hearts often show downregulation of PGC-1α and its downstream targets [43,44]. In spontaneously hypertensive rats (a genetic model of hypertension), left ventricular mitochondria have significantly lower PGC-1α levels, accompanied by depressed expression of regulators like sirtuin-1 and AMPK, which are normally protective and support biogenesis [43]. Thus, insufficient mitochondrial biogenesis in hypertension may exacerbate energy deficits.

### 4.4. Mitophagy

**Figure 1:**
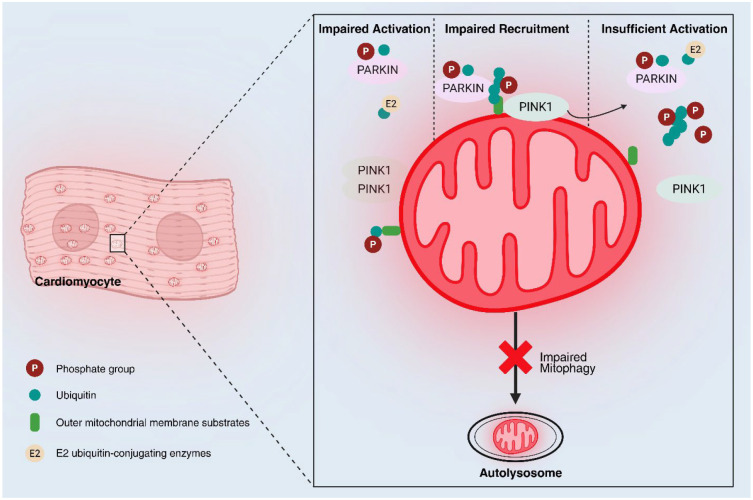
Impaired mitophagy in cardiomyocytes via dysregulated PINK1-Parkin pathway. Schematic representation of impaired mitophagy in a cardiomyocyte due to dysfunction in PINK1-Parkin pathway. Figure depicts a mitochondrion within a cardiomyocyte, highlighting molecular mechanisms involved. Impaired activation of PINK1 (PTEN-induced kinase 1) on the outer mitochondrial membrane is followed by impaired recruitment and insufficient activation of PARKIN, an E3 ubiquitin ligase. Phosphate groups (P) and ubiquitin (green dots) are shown, with outer mitochondrial membrane substrates (light green) and E2 ubiquitin-conjugating enzymes (yellow) participating in the process. Impaired PINK1-Parkin interaction causes insufficient ubiquitination and leads to failure of mitophagy, as indicated by the blocked pathway to the autophagosome, contributing to mitochondrial dysfunction and cardiomyocyte pathology.

Mitophagy is the selective autophagic degradation of damaged or superfluous mitochondria, a critical quality control mechanism. The best-characterized pathway is the PINK1/Parkin-mediated mitophagy. Under conditions of mitochondrial damage (e.g., loss of membrane potential), the kinase PINK1 accumulates on the outer membrane and recruits the E3 ubiquitin ligase Parkin from the cytosol. Parkin then ubiquitinates various outer membrane proteins, flagging the mitochondrion for enclosure by autophagosomes and degradation in lysosomes [45,46,47,48]. In the heart, basal mitophagy eliminates worn-out mitochondria, preventing accumulation of dysfunctional ROS-producing organelles [49,50]. Insufficient mitophagy can allow damaged mitochondria to persist and emit signals that cause inflammation or cell death [51]. Conversely, overly excessive mitophagy can deplete mitochondria and precipitate energy failure. Maintaining mitochondrial homeostasis requires coordinated biogenesis, dynamics, and mitophagy, with any imbalance potentially leading to disease. In cardiovascular disease including hypertension, evidence suggests mitophagy becomes dysregulated. For example, pressure overload-induced heart failure in mice is associated with reduced PINK1-Parkin mitophagy signaling, contributing to accumulation of defective mitochondria [52,53]. On the other hand, augmenting mitophagy has shown benefits in models of cardiac stress: one study found that enhancing Parkin-mediated mitophagy mitigated adverse remodeling after myocardial infarction, improving cardiac function [54]. In the context of hypertension, studies have implicated both PINK1/Parkin and receptor-mediated mitophagy pathways (e.g., BNIP3, FUNDC1) in hypertensive organ damage [55]. Notably, activating AMPK an energy-sensing kinase often suppressed in hypertension can stimulate protective mitophagy; AMPKα2 was shown to phosphorylate PINK1 and promote Parkin recruitment, preventing development of heart failure in a pressure-overload model [56] (see Figure 1).

Overall, the heart relies on efficient fusion to maintain mitochondrial function, fission to remove damaged parts, biogenesis to replenish the pool, and mitophagy to cull defective mitochondria. Hypertensive stress can disrupt each of these facets of mitochondrial dynamics, as discussed next. It is the interplay among these processes that determines whether a cardiomyocyte’s mitochondria adapt to stress or succumb to dysfunction.

## 5. Mechanisms of Mitochondrial Dysfunction in Hypertensive Heart Disease

In hypertensive heart disease, sustained high blood pressure and neurohormonal activation (e.g., renin–angiotensin–aldosterone system upregulation) create a hostile environment for mitochondria. A number of interrelated mechanisms drive mitochondrial dysfunction in the hypertensive heart, contributing to cellular energy shortfalls and tissue injury.

### 5.1. Structural Mitochondrial Alterations

Chronic hypertension causes ultrastructural remodeling of cardiac mitochondria. Electron microscopy studies in hypertensive models and patients have documented hallmark changes such as mitochondrial swelling, loss or disorganization of cristae, and fragmentation of the mitochondrial network [1,57,58]. Mitochondrial volume density in cardiomyocytes can decrease due to both a drop in biogenesis and possibly activation of mitophagy as a stress response [3,59]. The remaining mitochondria are often enlarged (“megamitochondria”) but paradoxically fewer in number, or sometimes abnormally small and fragmented, indicating a dysregulated fission/fusion balance. High blood pressure has also been associated with damage to cardiolipin, a phospholipid in the inner membrane that is critical for organizing cristae and respiratory chain supercomplexes [1,60]. Loss of cardiolipin impairs the structural integrity of cristae and the efficiency of electron transport. Together, these structural alterations—decreased mitochondrial number, swollen size, cristae remodeling or loss—have direct ramifications for function, as fewer and morphologically abnormal mitochondria produce less ATP and more ROS.

### 5.2. Bioenergetic Impairment

The hypertensive heart frequently exhibits reduced mitochondrial respiratory capacity. Isolated cardiac mitochondria from hypertensive animal models show depressed activities of electron transport chain (ETC) complexes and lower oxygen consumption rates (OCRs), a key parameter measured by Seahorse XF extracellular flux analysis [61,62] compared to normotensive controls [63,64]. The cristae abnormalities noted above (e.g., cristae rupture or simplification) likely underlie some of this respiratory inefficiency, because cristae architecture is crucial for optimal packing of ETC components. In a model of lipid overload simulating hypertension-related metabolic stress, cardiomyocytes exposed to palmitate displayed “abundant fragmented and lysed cristae” along with reduced expression of ETC complexes and a fragmented mitochondrial network [65]. This suggests that lipotoxicity (common in hypertensive patients with metabolic syndrome) can directly injure mitochondria, compounding the pressure overload stress.

Furthermore, mitochondrial oxidative phosphorylation substrates and preferences shift in hypertensive heart disease. As the myocardium hypertrophies and remodels, there is often a reversion to a more fetal metabolic profile increasing reliance on glucose and glycolysis and reduced fatty acid oxidation [66,67]. This shift, initially adaptive to maintain ATP production when mitochondria are impaired, eventually becomes maladaptive as it is less efficient in the adult heart. Indeed, in heart failure (frequently the end stage of HHD), metabolic substrate utilization changes contribute to mitochondrial dysfunction and impaired myocyte function. A chronically pressure-overloaded heart may have mitochondrial enzymatic deficiencies and altered expression of metabolic genes that prevent it from adequately using fatty acids, creating an energy-starved state despite plentiful fuel [68,69,70].

### 5.3. Oxidative Stress and ROS Signaling

Mitochondrial dysfunction in HHD leads to excess generation of ROS, such as superoxide and hydrogen peroxide. Hypertrophy and pressure overload are associated with increased activity of NADPH oxidases and xanthine oxidase, which raise baseline oxidative stress and can damage mitochondria [71,72,73]. In turn, damaged mitochondria (with ETC electron leak or impaired antioxidant defenses) produce even more ROS, establishing a vicious cycle. High ROS can oxidize mitochondrial DNA, proteins, and lipids like cardiolipin, further impairing respiration. Oxidative modification of adenine nucleotide translocator or other inner membrane proteins can also trigger the mitochondrial permeability transition pore (mPTP) to open, causing collapse of the membrane potential and cell death [26]. Angiotensin II, a key hypertensive hormone, is known to stimulate mitochondrial ROS production in cardiomyocytes [74,75]. Excessive mitochondrial fission in this setting may exacerbate ROS release, because fragmented mitochondria tend to be less bioenergetically efficient and more prone to generating ROS per unit mass [57,76,77]. Elevated ROS not only directly injure cells but also act as signaling molecules that provoke pathological remodeling; for instance, ROS activate matrix metalloproteinases and pro-fibrotic gene programs, contributing to interstitial fibrosis in HHD [78,79,80].

### 5.4. Excessive Fission and Insufficient Fusion

**Figure 2:**
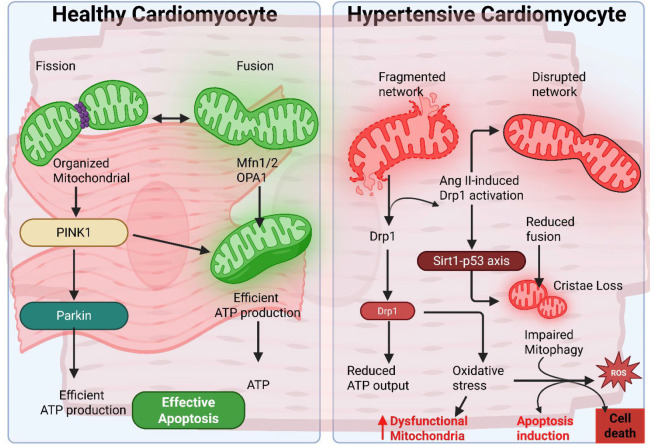
Mitochondrial dynamics in healthy and hypertensive cardiomyocytes. Left panel illustrates a healthy cardiomyocyte with an organized mitochondrial network maintained by balanced fission and fusion (mediated by Mfn1/2 and OPA1) and effective mitophagy via the PINK1-Parkin pathway, resulting in efficient ATP production and controlled apoptosis. Right panel shows a hypertensive cardiomyocyte with a fragmented and disrupted mitochondrial network due to Ang II-induced Drp1 activation through the Sirt1-p53 pathway, leading to excessive fission, decreased fusion, loss of cristae, and impaired mitophagy. This results in reduced ATP output, increased oxidative stress (ROS), dysfunctional mitochondria, and enhanced apoptosis, culminating in cell death.

Hypertensive stimuli can tilt the balance of mitochondrial dynamics toward fission. Angiotensin II has been shown to induce Drp1-mediated mitochondrial fission in cardiomyocytes, promoting cell death [81]. In one study, treating neonatal rat cardiomyocytes with Ang II increased Drp1 expression and mitochondrial fragmentation, while Drp1 inhibition (with the small molecule mdivi-1 or via gene silencing) attenuated the mitochondrial fission and protected the cells from hypertrophy and apoptosis [81]. In vivo, mice or rats subjected to Ang II infusion or pressure overload have demonstrated higher levels of active Drp1 and fragmented mitochondria in the myocardium, correlating with worse cardiac function. Conversely, genetic or pharmacological suppression of Drp1 can reduce hypertensive cardiac remodeling and dysfunction [81]. Mechanistically, Ang II triggers upstream signaling that impacts fission/fusion proteins; for example, Ang II was found to degrade the deacetylase Sirt1, leading to hyperacetylation of p53, which then upregulates Drp1 transcription, resulting in excessive fission and cardiomyocyte apoptosis [81]. This Sirt1-p53-Drp1 axis exemplifies how hypertensive signaling cascades can converge on mitochondrial dynamics pathways. On the fusion side, hypertension may reduce the expression of mitofusins and OPA1, or functionally impair them via post-translational modifications. Reduced Mfn2 in the heart has been reported under chronic stress, and OPA1 is sensitive to cleavage by OMA1, which is activated by mitochondrial membrane potential loss [57,82,83]. A consequence of fusion impairment is the loss of mitochondrial network connectivity and failure to compensate for localized damage, thereby compounding dysfunction. A summary of these disrupted dynamics is presented in Figure 2.

### 5.5. Impaired Mitophagy and Mitochondrial Turnover

Proper turnover of mitochondria is necessary to remove those that are irreversibly damaged. Hypertension appears to dysregulate mitophagy in complex ways. Some studies suggest that hypertrophic hearts have insufficient mitophagy; for instance, a transverse aortic constriction (TAC) model of pressure overload showed a decline in PINK1-Parkin signaling and accumulation of damaged mitochondria, contributing to contractile dysfunction [53]. Consistently, cardiomyocyte-specific Parkin knockout mice develop exacerbated hypertensive cardiomyopathy, whereas Parkin overexpression or autophagy-enhancing treatments (like spermidine) can ameliorate cardiac hypertrophy and dysfunction in hypertension [55,84,85]. Spermidine, which stimulates autophagy/mitophagy, was found to prevent the progression of heart failure in salt-sensitive hypertensive rats, coincident with lowered blood pressure and regression of left ventricular hypertrophy [55,84,85]. These findings indicate that enhancing mitochondrial quality control can counteract some deleterious effects of hypertension on the heart. On the other hand, certain contexts might feature excessive mitophagy contributing to mitochondrial loss. A delicate balance is evident: for example, knockout of the mitophagy receptor BNIP3 in mice attenuates cardiac fibrosis and dysfunction in pressure overload, implying that unrestrained BNIP3-mediated mitophagy might worsen cell death [86,87]. Regional differences within the cell (perinuclear mitochondria might be turned over differently than subsarcolemmal ones) and temporal changes make this a complex picture. Nonetheless, a consistent theme is that failing hypertensive hearts often have an accumulation of abnormal mitochondria that the cell has failed to clear, indicating a relative insufficiency of effective mitophagy.

### 5.6. Calcium Handling and mPTP Activation

**Figure 3:**
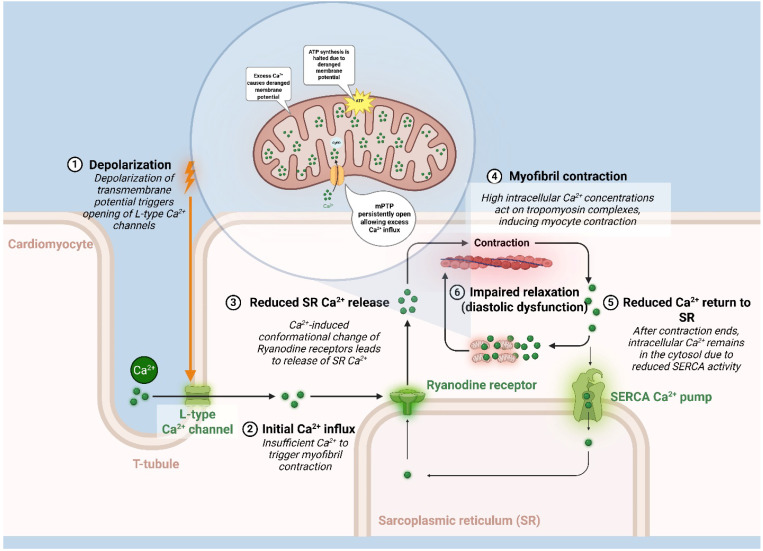
Dysregulated calcium handling in cardiomyocyte pathophysiology. This is a schematic representation of calcium (Ca^2+^) handling dysregulation in a cardiomyocyte under pathological conditions. (1) Depolarization of the transmembrane potential triggers the opening of L-type Ca**^2+^** channels. (2) Initial Ca**^2+^** influx is insufficient to trigger myofibril contraction. (3) Reduced sarcoplasmic reticulum (SR) Ca**^2+^** release due to Ca**^2+^**-induced conformational change of ryanodine receptors. (4) Myofibril contraction occurs with high intracellular Ca**^2+^** concentrations acting on troponin–myosin complexes. (5) Reduced Ca**^2+^** return to SR after contraction ends due to decreased sarco/endoplasmic reticulum Ca**^2+^**-ATPase (SERCA) activity. (6) Impaired relaxation (diastolic dysfunction) results from persistent Ca**^2+^** in the cytosol, exacerbated by mitochondrial permeability transition pore (mPTP) opening and halted ATP synthesis due to deranged membrane potential.

Hypertensive heart disease is typified by diastolic dysfunction, partly due to impaired calcium handling (e.g., reduced SERCA uptake, elevated diastolic Ca^2+^). Mitochondria in a hypertensive cardiomyocyte may experience chronically elevated calcium influx [88,89]. Excess matrix Ca^2+^ can precipitate the opening of the mitochondrial permeability transition pore. Once the mPTP opens persistently, the mitochondrion can no longer maintain its membrane potential, halting ATP synthesis and releasing pro-apoptotic factors. Even transient openings of mPTP (which can occur during calcium overload or oxidative stress) dissipate mitochondrial proton motive force and can contribute to arrhythmogenic events and contractile dysfunction. Normally, cyclophilin D and other components tightly regulate mPTP opening; however, oxidative stress and high Ca^2+^ in hypertension tilt the balance toward pore opening [90]. In parallel, decreased ATP output from sick mitochondria impairs the myocyte’s ATP-dependent calcium pumps, worsening cytosolic Ca^2+^ control a feed-forward loop that damages both excitation–contraction coupling and mitochondria themselves. This pathological cycle of calcium dysregulation is illustrated in Figure 3.

### 5.7. Inflammation and Mitochondria-Immune Crosstalk

Chronic inflammation is increasingly recognized in hypertensive heart disease and heart failure [14]. Mitochondrial dysfunction can be both a source and a consequence of inflammation. Damaged mitochondria may release danger-associated molecular patterns (DAMPs) such as mitochondrial DNA into the cytosol or circulation, which activate inflammatory pathways (e.g., via Toll-like receptors or the NLRP3 inflammasome). In hypertensive models, episodes of mitochondrial permeability transition and necrosis can incite sterile inflammation in the myocardium [91,92]. Conversely, cytokines and inflammatory cell infiltrates can produce nitric oxide and other factors that inhibit mitochondrial respiration and disrupt dynamics. Mitochondria-ER contact sites also appear to be altered in heart failure, affecting calcium and inflammatory signaling crosstalk [14]. The cumulative effect is a downward spiral where mitochondrial dysfunction and inflammation reinforce each other, leading to myocardial fibrosis and dysfunction [14].

In summary, hypertensive heart disease generates a convergence of damaging factors for mitochondria, such as structural damage to the organelles, suppressed biogenic renewal, imbalanced fission–fusion favoring fragmentation, defective removal of bad mitochondria, metabolic fuel shifts, and a high oxidative and calcium stress milieu. All these factors interconnect; for example, excessive fission yields fragmented mitochondria that produce more ROS, which in turn oxidize mitochondrial proteins and DNA, further lowering respiratory performance and signaling for cell death. Notably, many antihypertensive therapies (ACE inhibitors, beta-blockers) indirectly improve mitochondrial health by reducing the workload and neurohormonal drive on the heart [93,94]. However, these do not directly reverse mitochondrial damage. There is, therefore, considerable interest in mitochondria-targeted interventions for HHD. Antioxidants targeted to mitochondria (like mitoQ), agents that inhibit excessive fission (Drp1 inhibitors), or compounds that stimulate mitochondrial biogenesis (AMPK activators, PGC-1α agonists) are being explored in preclinical studies [95,96]. The goal of such therapies is to break the pathogenic cycle of mitochondrial dysfunction in the hypertensive heart and thereby halt or reverse cardiac remodeling.

## 6. Age-Related Mitochondrial Changes in the Heart

**Figure 4:**
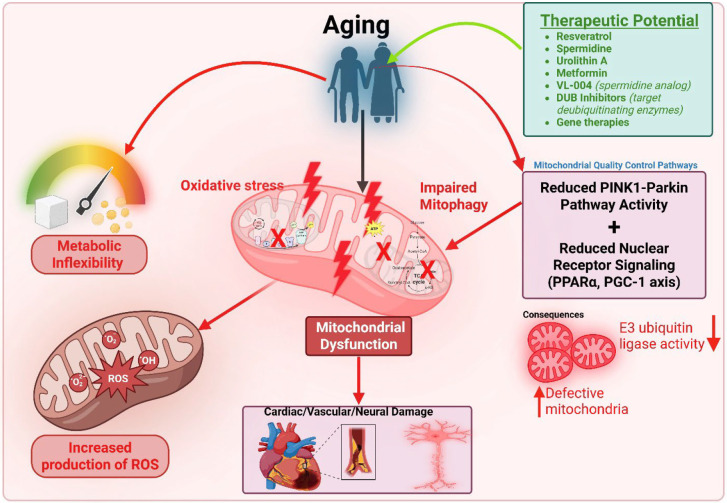
Aging-induced mitochondrial dysfunction with systemic effects on cardiovascular health and therapeutic potential. Aging promotes metabolic inflexibility and reduces the activity of key mitochondrial quality control pathways, including the PINK1-Parkin pathway and nuclear receptor signaling (e.g., PPARα, PGC-1), leading to impaired mitophagy and mitochondrial dysfunction. This dysfunction, exacerbated by aging, results in increased production of reactive oxygen species (ROS) due to oxidative stress and a detrimental feedback loop involving protein complexes that worsen mitochondrial damage. As illustrated, this cascading impact affects multiple organs: cardiac damage (depicted as a heart with a dark patch), vascular issues (shown as a narrowed blood vessel), and neurological damage (represented by a degenerating neuron). These manifestations contribute to the development of cardiovascular diseases (CVDs) such as stroke, ischemic heart disease, and heart failure, highlighting the critical link between mitochondrial health, aging, and systemic disease.

Cardiac aging is accompanied by a progressive decline in mitochondrial form and function, which has implications for hypertensive heart disease typically presenting later in life. Even in the absence of overt disease, aged hearts show several mitochondrial alterations compared to young hearts. These age-associated changes include: reduced mitochondrial density and ATP-generating capacity, increased production of ROS, accumulation of mitochondrial DNA mutations, and changes in the expression of fission/fusion and biogenesis regulators [10,14]. Collectively, these changes can render the myocardium of an elderly hypertensive patient less resilient to stress. The systemic impact of aging on mitochondrial health and cardiovascular disease is illustrated in Figure 4.

One striking age-related change is in mitochondrial ultrastructure. A recent 3D electron microscopy study quantified how cardiac mitochondria in mice morphologically evolve from young adulthood to advanced age [12]. From 3 months to 2 years of age (a rough equivalent of a human moving from young adult to senior), murine cardiac mitochondria exhibited a loss of cristae density and derangement of cristae morphology across aging, accompanied by a decrease in three-dimensional mitochondrial volume. In essence, aged cardiomyocytes had smaller, less complex mitochondria than their younger counterparts. These structural deficits mirror changes seen when key cristae maintenance proteins are disrupted: the same study noted that knocking out MICOS complex components (like Mitofilin, CHCHD3/6) or the fusion protein OPA1 in cardiac cells led to mitochondria with reduced volume and length and poorer respiratory function [12]. This parallel suggests that aging may involve a partial loss-of-function of mitochondrial fusion machinery and cristae organizers. Indeed, levels of OPA1 have been reported to decline with age in the heart, and long-OPA1 (the fusogenic form) is cleaved into short fragments more readily in aged mitochondria, which could explain the cristae loss. Similarly, components of the MICOS complex might become less abundant or less functional with age, leading to the observed cristae fenestration and flattening [12].

Aged hearts also tend to have a more fragmented mitochondrial network, reflecting an imbalance between fission and fusion [14]. In aged mouse cardiomyocytes, there is often increased Drp1 activity/localization on mitochondria and reduced mitofusin levels, tipping the scale toward fission [97]. Fragmentation, coupled with the drop in total mitochondrial volume, may significantly reduce the heart’s energy reserve. Moreover, aging impairs mitophagy efficiency. Studies have shown that older hearts accumulate mitochondria with deletions in mtDNA and evidence of oxidative damage that would normally be cleared by mitophagy [98,99,100]. The PINK1-Parkin pathway activity can decline with age, partly due to lower Parkin levels or impaired autophagosome formation in aged cells. This results in retention of dysfunctional mitochondria in cardiomyocytes of older individuals. For example, hearts of aged mice display higher oxidative modification of mitochondrial proteins and lipid peroxidation despite upregulation of some antioxidant enzymes, indicating that damaged mitochondria are persisting [101,102]. Transgenic augmentation of mitochondrial catalase or other antioxidants in mice has been shown to reduce mtDNA damage and preserve cardiac function with aging [103,104,105], supporting the idea that intrinsic mitochondrial deterioration is a driver of cardiac aging.

Importantly, the aging process influences substrate metabolism in the heart. An aged heart becomes less flexible in its metabolic switch, often showing blunted capacity to increase fatty acid oxidation when needed and a greater reliance on glucose at baseline, somewhat akin to a diabetic heart. This “metabolic inflexibility” might stem from accumulated mitochondrial DNA damage, impairing electron transport enzymes for fatty acid β-oxidation. Additionally, an age-related decline in nuclear receptor signaling (e.g., PPARα/PGC-1α axis) reduces the transcription of mitochondrial oxidative enzymes [106]. The net effect is that an aged myocardium has a lower oxidative phosphorylation reserve. Under hypertensive stress, such a heart may decompensate faster because it cannot adequately upregulate mitochondrial function or replace damaged mitochondria.

Interactions between aging and hypertension on mitochondria are an area of active research. Evidence suggests a synergistic detriment: for instance, aged hypertensive animals have a dramatically higher burden of cardiac mtDNA mutations and oxidative damage than either aged normotensive or young hypertensive counterparts. Also, aged hypertensive hearts show earlier onset of diastolic dysfunction, potentially related to mitochondrial Ca^2+^ handling issues. A pertinent study by Zer Vue and colleagues (2023) demonstrated that age-related mitochondrial structural changes in mice (cristae loss, smaller mitochondria) were remarkably similar to those induced by genetic disruption of MICOS complex proteins [12]. Since MICOS deficiencies cause both mitochondrial and cardiac contractile defects [107], this raises the hypothesis that an age-linked decline in MICOS integrity in the heart might be one factor making aged mitochondria more vulnerable to hypertensive stress. Additionally, older hearts likely have diminished stress responses; for example, reduced AMPK activation under pressure overload, leading to insufficient mitophagy and biogenesis when faced with hypertension.

**Figure 5:**
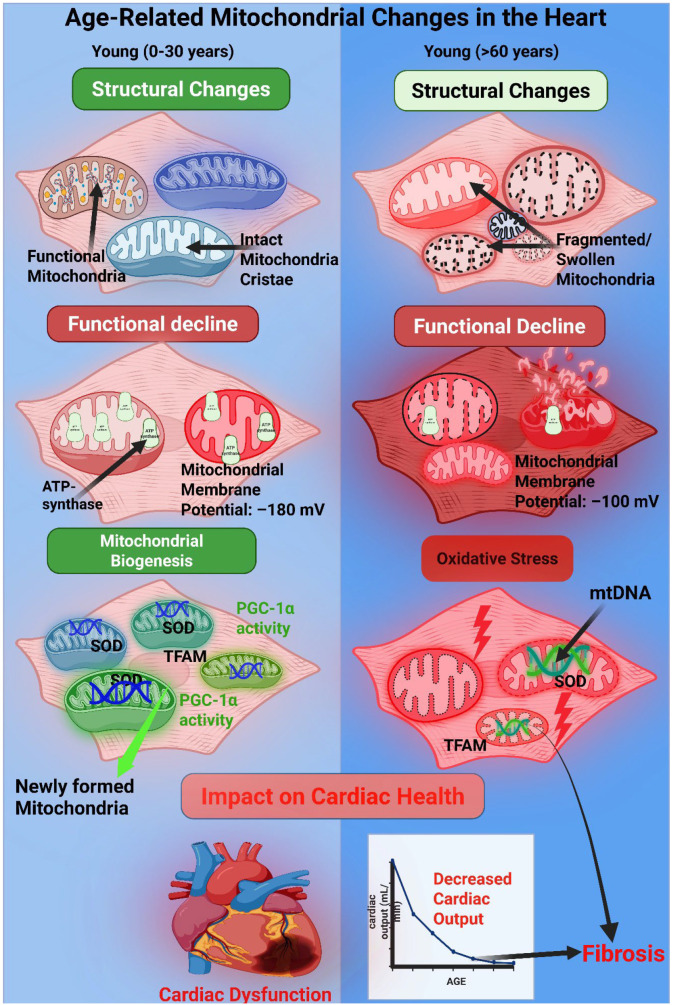
Age-related mitochondrial alterations in the heart and their impact on cardiac health. This schematic compares mitochondrial structure and function in cardiac tissue between young (0–30 years) and elderly (>60 years) individuals. In the young heart (left panel), mitochondria exhibit intact cristae, high ATP synthase density, optimal membrane potential (−180 mV), low reactive oxygen species (ROS), and robust mitochondrial biogenesis driven by PGC-1α, SOD2, and TFAM. In contrast, aged cardiomyocytes (right panel) show fragmented and swollen mitochondria, reduced ATP synthase expression, diminished membrane potential (−100 mV), increased oxidative stress with mtDNA damage and lipid peroxidation, and impaired biogenesis. These age-related mitochondrial deficits are associated with decreased cardiac output and increased fibrosis, which contribute to progressive cardiac dysfunction.

Thus, in hypertensive heart disease, patient age is a crucial modifier of mitochondrial pathology. The aging heart brings to the table a set of pre-existing mitochondrial compromises: accumulated damage, a less interconnected network, and progressively impaired quality control. These exacerbate the mitochondrial dysfunction triggered by hypertension. Clinically, this may translate into older hypertensive patients being more prone to heart failure with preserved ejection fraction (HFpEF), where diastolic dysfunction and energetic deficit are key, as well as reduced recovery capacity after episodes of acute decompensation. An appreciation of age-related mitochondrial changes, therefore, informs the need for age-specific therapeutic strategies; for instance, interventions to boost mitophagy or biogenesis might particularly benefit older hypertensive individuals. A comparative schematic of the key mitochondrial alterations in the young versus aged heart is provided in Figure 5.

## 7. Recent Research Advances

Research in the past few years has significantly advanced our understanding of mitochondrial dynamics and dysfunction in hypertensive heart disease, thanks to new technologies and interdisciplinary approaches. Notably, both animal studies and human tissue analyses have provided fresh insights that could lead to novel therapies. In this section, we highlight some key recent advances and how they inform the conceptual framework described above.

### 7.1. Ultrastructural Imaging of Cardiac Mitochondria

Traditional 2D electron microscopy has long hinted at mitochondrial abnormalities in disease, but recent studies using three-dimensional electron microscopy techniques (such as serial block-face scanning EM and electron tomography) allow a far more quantitative and nuanced view. Vue et al. [12] performed 3D reconstructions of cardiac mitochondria in mice across the lifespan. This study was the first to quantify age-associated mitochondrial network changes in the heart, confirming a clear decline in mitochondrial volume and cristae structure with aging, as discussed. Extending this approach to disease, Vue and colleagues very recently applied 3D EM to failing human hearts [13]. In a 2023 bioRxiv preprint, they reported that human heart failure (with both ischemic and non-ischemic etiologies) is accompanied by distinct structural alterations in mitochondria and myofibrils [13]. Mitochondria from failing hearts showed shape changes and likely volume differences compared to non-failing donor hearts, while myofibrils in failing hearts had increased cross-sectional area and branching [13]. These structural perturbations were correlated with shifts in metabolomic and lipidomic profiles [13], suggesting that the physical disruption of mitochondrial architecture in heart failure might be mechanistically linked to metabolic remodeling. Although that study did not focus specifically on hypertensive heart disease, many heart failure patients have a history of hypertension; thus, the findings are relevant. They underscore how personalized structural phenotyping of mitochondria (e.g., identifying a patient’s specific pattern of mitochondrial enlargement or loss) might guide tailored interventions in the future.

### 7.2. Regulators of Mitochondrial Network Organization

A breakthrough in fundamental mitochondrial biology came from a Nature Communications study by Katti et al. [11], which identified evolutionarily conserved regulators of muscle mitochondrial network organization. By leveraging *Drosophila* genetics, the study uncovered over 140 proteins associated with distinct muscle mitochondrial phenotypes (either tightly packed parallel networks or more reticular grid-like networks). Intriguingly, they showed that certain transcription factors, notably cut, salmon (salm), and H15, can independently regulate mitochondrial network configuration separate from muscle fiber type. For example, loss of salm converted the normally parallel mitochondrial networks in fly flight muscle into a grid-like arrangement akin to leg muscle, without changing the muscle’s contractile protein type. These transcriptional regulators have mammalian homologs that may play roles in cardiac muscle. The implication is that the heart’s mitochondrial network (which in healthy myocardium appears as a regular lattice of individual organelles between myofibrils) might be actively maintained by specific gene programs and in disease, these programs could be altered. While direct ties to hypertension are not yet made, one could speculate that hypertrophic signaling pathways might influence such network regulators. The concept of independent control of mitochondrial network geometry and content is a new paradigm; it means we might modulate how mitochondria are arranged and connected in heart cells without necessarily changing their number, potentially affecting efficiency and resilience. This is an area ripe for exploration in hypertensive models.

### 7.3. Role of the MICOS Complex in Cardiac Health

The MICOS has emerged as a critical player in cardiac mitochondrial integrity. As mentioned, Vue et al. [12] pointed to the MICOS decline in aging. In parallel, a 2023 study by Birker et al. revealed the importance of MICOS genes in heart development and disease [107]. This study was motivated by genetic findings in hypoplastic left heart syndrome (HLHS), a congenital defect, where variants in MICOS complex genes were implicated. Using Drosophila and mouse models, the researchers showed that partial loss of MICOS components (like CHCHD3/6, which are Mic19/Mic25 in the complex) in the heart led to drastically impaired contractility, sarcomere disarray, reduced ATP levels, and combined mitochondrial fission–fusion defects. In essence, MICOS disruption phenocopied severe cardiomyopathy. They further demonstrated that these effects were similar to knocking down ATP synthase subunits, underlining how cristae destabilization can depress energy production and muscle function. The same study also found that Opa1 (inner membrane fusion protein) interacts functionally with MICOS: Opa1 knockdown in fly hearts caused contractile dysfunction and arrhythmia, and combined Opa1+MICOS deficiencies worsened outcomes. The recognition that the MICOS complex is necessary to maintain cristae morphology and ATP production in heart mitochondria places an even greater emphasis on how cristae structure perturbations contribute to hypertensive heart disease. It is conceivable that chronic hypertensive stress or aging might downregulate MICOS components or alter their function (for instance, via post-translational modifications from ROS), leading to the cristae aberrations observed in HHD. Ongoing research is examining MICOS integrity in failing human hearts. From a therapeutic standpoint, stabilizing cristae structure (perhaps by preventing MICOS loss or enhancing OPA1 activity) might preserve mitochondrial function in hypertension.

### 7.4. Mitochondria and Heart Failure Phenotypes

Antentor Hinton Jr. and colleagues published a series of papers in 2023–2024 that deepen our understanding of mitochondria in cardiac disease. In a comprehensive 2024 *Circulation Research* review, Hinton’s group emphasized how mitochondrial structural and functional changes contribute to human heart failure, and they spotlighted often-neglected factors such as mitochondria-ER contact sites and mitochondrial nanotunnels in cardiac pathology [14]. They note that mitochondrial morphology is closely linked to function, changing via dynamics to meet cellular energy needs, and that in heart failure, this relationship is perturbed. One intriguing phenomenon is the appearance of “donut-shaped” mitochondria and other unusual morphologies in stressed hearts. Although the significance of donut mitochondria (mitochondria that have folded into a torus-like shape) is not fully clear, they have been correlated with high oxidative stress and possibly represent an adaptive attempt to maintain membrane potential under stress [14]. Hinton’s group also discusses how chronic inflammation in heart failure interacts with mitochondrial dysfunction, creating a cycle of damage [14]. This is particularly relevant to hypertensive heart failure, where low-grade inflammation often accompanies hypertension (due to vascular oxidative stress, etc.). Additionally, a 2024 study by Jenkins et al. (co-authored by Hinton) explores “mitochondria in disease: changes in shapes and communication,” indicating a growing interest in how changes like increased mitochondrial networking or tunneling might occur in disease states [108]. These works collectively drive home that in conditions like HHD that progress to heart failure, we must consider not just classic bioenergetics, but also the organelle’s social network how mitochondria interact with each other and with other organelles.

### 7.5. Insights from Exercise and Epigenetics

While not directly about hypertension, research on exercise offers relevant clues since exercise is known to enhance mitochondrial quality. Murach et al. [109] and others have studied how exercise training impacts muscle mitochondria, showing that physiological hypertrophy (from exercise) boosts mitochondrial biogenesis and respiratory function, in stark contrast to pathological hypertrophy from hypertension. For example, exercise triggers PGC-1α and NRF1 upregulation, increases mitochondrial content, and improves cristae density in the heart. These findings underscore that not all hypertrophy is equal; the molecular context (endocrine environment, hemodynamics) matters. The hope is to coax a pathological hypertrophied heart to adopt some of the mitochondrial benefits of exercise. Indeed, therapies like AMPK activators or PPAR agonists attempt to mimic aspects of the exercise response. Another angle is epigenetics: chronic hypertension can alter the expression of nuclear genes controlling mitochondria via DNA/histone modifications. Restoring youthful expression patterns of mitochondrial regulators (perhaps via deacetylase activators like SIRT1 agonists) may prove fruitful. A recent study in Aging Cell [110] showed that an 8-week treatment with the mitochondria-targeted peptide elamipretide in aged mice improved cardiac function (increasing cardiac strain and diastolic function) and skeletal muscle performance. Elamipretide aids mitochondrial electron transport and combats ROS. Interestingly, these functional gains occurred without changes in “tissue epigenetic or transcriptomic age,” implying that even if the overall gene expression profile remains aged, directly supporting mitochondrial function pharmacologically can confer benefits. Extrapolating to HHD, such agents might improve an aged hypertensive heart’s resilience.

### 7.6. The Translational Potential of SGLT2 Inhibitors

Notably, sodium-glucose cotransporter 2 (SGLT2) inhibitors, a class of drugs with proven mortality benefits in heart failure, have been increasingly linked to improved mitochondrial function. While their primary action is glycosuric, their cardioprotective effects are attributed to pleiotropic mechanisms, including a shift in myocardial fuel utilization towards ketones and a more efficient energy substrate, reduction in ventricular load, and direct improvements in mitochondrial bioenergetics and reduction of oxidative stress [111,112]. Their clinical success underscores the therapeutic principle of targeting metabolic and mitochondrial pathways in hypertensive heart failure, providing a strong rationale for developing next-generation, direct mitochondrial therapeutics.

### 7.7. Therapeutic Targeting of Mitochondrial Dynamics

Building on mechanistic insights, there have been experimental forays into drugs targeting dynamics. We mentioned mdivi-1 (a Drp1 inhibitor), which in rodent studies reduced Ang II-induced hypertension, cardiac hypertrophy, and fibrosis [113]. Another compound, SS-31 (also known as Bendavia, a mitochondrial-targeted peptide), has been shown to preserve cristae curvature and improve efficiency of oxidative phosphorylation; in models of hypertension or myocardial infarction, SS-31-treated animals had better cardiac function and less hypertrophy. Gene therapy or peptide delivery to boost OPA1 or mitofusin levels in the heart is being explored in preclinical models of heart failure, with promising results in normalizing mitochondrial structure. Furthermore, mitophagy enhancers like spermidine (which can be obtained through dietary supplementation) or urolithin A are under investigation for their cardioprotective effects in aging and hypertensive models. Cumulatively, these advances illustrate a translational trend: researchers are moving beyond correlative studies and actively testing interventions on mitochondrial targets a necessary step if we are to translate mitochondrial biology into treatments for hypertensive patients.

Finally, a noteworthy development is the improvement in diagnostic imaging and biomarkers for mitochondrial dysfunction. For instance, magnetic resonance spectroscopy can noninvasively measure cardiac phosphocreatine/ATP ratios, serving as a proxy for mitochondrial function in patients [114]. Hypertensive patients with heart failure have reduced ratios, and improvement in this metric correlates with therapy response. There is also growing interest in circulating mitochondrial DNA as a biomarker of cardiac stress. Hypertensive individuals show elevated cell-free mtDNA during acute hypertension episodes, reflecting tissue mitochondrial damage. As research progresses, we may see a panel of mitochondrial biomarkers used clinically to identify which hypertensive patients are at the highest risk of progressing to heart failure, thus prompting early aggressive therapy.

In summary, the recent few years have seen leaps in our understanding: from high-resolution mapping of mitochondrial changes (spatial and temporal), identification of new molecular players (MICOS, network regulators), to proof-of-concept therapies targeting mitochondrial dynamics. These advances paint a hopeful picture wherein, rather than viewing mitochondrial damage in HHD as an inexorable consequence, we might intervene to correct or mitigate it. The challenge moving forward is to integrate these findings into a cohesive strategy to treat or even prevent hypertensive heart disease by preserving mitochondrial health.

## 8. Therapeutic Implications and Future Directions

The accumulating evidence linking mitochondrial dysfunction to HHD progression has paved the way for novel therapeutic strategies aimed at preserving or restoring mitochondrial health. While standard antihypertensive therapies provide hemodynamic relief and indirectly improve mitochondrial function by reducing cardiac workload, direct mitochondrial-targeted interventions represent a promising frontier for halting or reversing maladaptive remodeling. Key pathological mechanisms and their corresponding potential therapeutic approaches are summarized in Table 1.

## 9. Conclusions

Mitochondrial dysfunction and deranged dynamics lie at the heart of hypertensive cardiac pathology. Chronic hypertension imposes a relentless energy demand and oxidative strain on cardiomyocytes, and the mitochondria respond by undergoing structural remodeling, functional decline, and altered quality control changes that feed forward to exacerbate cardiac damage. We have seen that in hypertensive heart disease, mitochondria become fewer and structurally abnormal with swollen profiles and fragmented cristae, leading to reduced ATP output and increased ROS production [1]. Hypertensive signaling pathways (e.g., Ang II/p53, adrenergic stress) can directly provoke excessive mitochondrial fission and insufficient fusion, tipping the balance toward a fragmented network prone to dysfunction [81]. At the same time, the hypertrophied heart often fails to adequately upregulate mitochondrial biogenesis and mitophagy, resulting in an accumulation of dysfunctional mitochondria that further impair cellular function. The compounding factor of age adds further stress, as aging hearts already exhibit cristae degeneration and weakened dynamic responses [12]. Together, these factors create an energetic deficit and pro-death milieu that drives HHD toward heart failure.

Encouragingly, the latest research offers reasons for optimism. The identification of critical players in mitochondrial ultrastructure maintenance (like MICOS and OPA1) and regulators of network configuration opens new therapeutic targets; for example, stabilizing cristae architecture or modulating specific gene pathways to restore healthy mitochondrial networks in the failing heart. Already, in experimental models, interventions such as Drp1 inhibition, AMPK activation, or autophagy enhancement can attenuate hypertensive cardiac injury, underscoring that mitochondrial pathology is to an extent, reversible or preventable. Clinically, while standard antihypertensive treatments reduce the load on the heart and secondarily benefit mitochondria [1], future treatments might act directly on cardiomyocyte mitochondria. Trials of mitochondrial-targeted antioxidants, peptide therapeutics (like elamipretide), and metabolic modulators in heart failure are underway and may eventually be applied specifically to patients with HHD.

Another emerging concept is personalized mitochondrial medicine. As advanced imaging and omics technologies become more accessible, clinicians may detect specific mitochondrial deficits in a patient’s heart (for instance, a pronounced fusion defect vs. a primary issue of mitophagy failure). This could guide tailored therapies one patient might benefit most from a fusion promoter, while another from a mitophagy booster. The integrated structural and metabolomic analyses of failing human hearts [13] represent the first steps in this direction, revealing heterogeneity in mitochondrial phenotypes that could be leveraged for personalized intervention.

In conclusion, mitochondria are both hapless victims and active instigators in hypertensive heart disease. Their dysfunction is a unifying thread that links mechanical stress to molecular injury within cardiomyocytes. By unraveling the mechanisms of mitochondrial dynamics and damage in HHD, we not only improve our understanding of disease progression but also illuminate novel avenues for therapy. Keeping the “flame” of the heart’s powerhouse alive and efficient may prove key to preventing the transition from compensated hypertensive hypertrophy to decompensated heart failure. The recent advances summarized in this review herald a paradigm shift from treating hypertension’s hemodynamic numbers to treating the cellular power plants that truly drive cardiac health. As research continues, a future in which clinicians can monitor and pharmaceutically optimize cardiac mitochondrial health in hypertensive patients appears increasingly within reach, offering hope to millions at risk for hypertensive heart disease and its dire complications.

## Figures and Tables

**Table 1:** Summary of key mitochondrial derangements in hypertensive heart disease and emerging therapeutic strategies.

Mitochondrial Derangement	Mechanistic Drivers in HHD	Functional Consequences	Potential Therapeutic Strategies and Agents
Excessive Fission	Ang II → Sirt1/p53 → Drp1 activation; reduced Mfn2	Network fragmentation, ROS overproduction, apoptosis	Drp1 inhibition: Mdivi-1 [81,112]
Impaired Fusion	Reduced Mfn1/2; OPA1 cleavage via OMA1	Loss of complementation, cristae disassembly, and energetic deficiency	Fusion promotion: Mfn2/OPA1 gene therapy; OMA1 inhibition [57,82]
Cristae Disruption	MICOS complex dysfunction; cardiolipin damage	Impaired ETC supercomplex assembly, reduced OXPHOS efficiency	Cristae stabilization: Elamipretide (SS-31); targeting MICOS components [12,107]
Defective Mitophagy	Impaired PINK1/Parkin signaling; BNIP3 dysregulation	Accumulation of damaged mitochondria, inflammation	Mitophagy enhancers: Spermidine; Urolithin A; AMPK activators [55,56,84,85]
Oxidative Stress	ETC leak; NOX/XO activation; antioxidant deficit	mtDNA/protein/lipid damage, mPTP opening, signaling dysfunction	Mitochondrial antioxidants: MitoQ; SkQ1 [95,96]
Biogenesis Impairment	Reduced PGC-1α/NRF1 signaling; Sirt1/AMPK inhibition	Inadequate mitochondrial renewal, energy deficit	Biogenesis induces: AMPK activators (e.g., Metformin); PPARα agonists [40,41,42,43,90,93]

Abbreviations: HHD: hypertensive heart disease; Ang II: angiotensin II; Drp1: dynamin-related protein 1; Mfn: mitofusin; OPA1: optic atrophy 1; MICOS: mitochondrial contact site and cristae organizing system; ETC: electron transport chain; OXPHOS: oxidative phosphorylation; PINK1: PTEN-induced kinase 1; ROS: reactive oxygen species; mPTP: mitochondrial permeability transition pore; PGC-1α: peroxisome proliferator-activated receptor gamma coactivator 1-alpha; NRF1: nuclear respiratory factor 1; AMPK: AMP-activated protein kinase.

## References

[1] Eirin A., Lerman A., Lerman L.O. (2014). Mitochondrial Injury and Dysfunction in Hypertension-Induced Cardiac Damage. Eur. Heart J..

[2] Wang Y., Wang Y., Zhang W. (2025). Dysregulation of Mitochondrial in Pulmonary Hypertension-Related Right Ventricular Remodeling: Pathophysiological Features and Targeting Drugs. Rev. Cardiovasc. Med..

[3] Zong Y., Li H., Liao P., Chen L., Pan Y., Zheng Y., Zhang C., Liu D., Zheng M., Gao J. (2024). Mitochondrial Dysfunction: Mechanisms and Advances in Therapy. Signal Transduct. Target. Ther..

[4] Chen L., Gong Q., Stice J.P., Knowlton A.A. (2009). Mitochondrial OPA1, Apoptosis, and Heart Failure. Cardiovasc. Res..

[5] Hom J., Yu T., Yoon Y., Porter G., Sheu S.-S. (2010). Regulation of Mitochondrial Fission by Intracellular Ca^2+^ in Rat Ventricular Myocytes. Biochim. Biophys. Acta.

[6] Kindo M., Gerelli S., Bouitbir J., Charles A.-L., Zoll J., Hoang Minh T., Monassier L., Favret F., Piquard F., Geny B. (2012). Pressure Overload-Induced Mild Cardiac Hypertrophy Reduces Left Ventricular Transmural Differences in Mitochondrial Respiratory Chain Activity and Increases Oxidative Stress. Front. Physiol..

[7] Ravindran S., Rau C.D. (2024). The Multifaceted Role of Mitochondria in Cardiac Function: Insights and Approaches. Cell Commun. Signal..

[8] Chistiakov D.A., Sobenin I.A., Revin V.V., Orekhov A.N., Bobryshev Y.V. (2014). Mitochondrial Aging and Age-Related Dysfunction of Mitochondria. Biomed. Res. Int..

[9] Somasundaram I., Jain S.M., Blot-Chabaud M., Pathak S., Banerjee A., Rawat S., Sharma N.R., Duttaroy A.K. (2024). Mitochondrial Dysfunction and Its Association with Age-Related Disorders. Front. Physiol..

[10] Tocchi A., Quarles E.K., Basisty N., Gitari L., Rabinovitch P.S. (2015). Mitochondrial Dysfunction in Cardiac Aging. Biochim. Biophys. Acta (BBA)-Bioenerg..

[11] Katti P., Ajayi P.T., Aponte A., Bleck C.K.E., Glancy B. (2022). Identification of Evolutionarily Conserved Regulators of Muscle Mitochondrial Network Organization. Nat. Commun..

[12] Vue Z., Neikirk K., Vang L., Garza-Lopez E., Christensen T.A., Shao J., Lam J., Beasley H.K., Marshall A.G., Crabtree A. (2023). Three-Dimensional Mitochondria Reconstructions of Murine Cardiac Muscle Changes in Size across Aging. Am. J. Physiol.-Heart Circ. Physiol..

[13] Vue Z., Ajayi P.T., Neikirk K., Murphy A.C., Prasad P., Jenkins B.C., Vang L., Garza-Lopez E., Mungai M., Marshall A.G. (2023). Human Heart Failure Alters Mitochondria and Fiber 3D Structure Triggering Metabolic Shifts. bioRxiv.

[14] Hinton A., Claypool S.M., Neikirk K., Senoo N., Wanjalla C.N., Kirabo A., Williams C.R. (2024). Mitochondrial Structure and Function in Human Heart Failure. Circ. Res..

[15] Watson W.D., Arvidsson P.M., Miller J.J.J., Lewis A.J., Rider O.J. (2024). A Mitochondrial Basis for Heart Failure Progression. Cardiovasc. Drugs Ther..

[16] Maack C., Cortassa S., Aon M.A., Ganesan A.N., Liu T., O’Rourke B. (2006). Elevated Cytosolic Na+ Decreases Mitochondrial Ca^2+^ Uptake During Excitation-Contraction Coupling and Impairs Energetic Adaptation in Cardiac Myocytes. Circ. Res..

[17] Balaban R.S., Bose S., French S.A., Territo P.R. (2003). Role of Calcium in Metabolic Signaling between Cardiac Sarcoplasmic Reticulum and Mitochondria in Vitro. Am. J. Physiol. Cell Physiol..

[18] Territo P.R., Mootha V.K., French S.A., Balaban R.S. (2000). Ca^2+^ Activation of Heart Mitochondrial Oxidative Phosphorylation: Role of the F(0)/F(1)-ATPase. Am. J. Physiol. Cell Physiol..

[19] Mailloux R.J., Jin X., Willmore W.G. (2014). Redox Regulation of Mitochondrial Function with Emphasis on Cysteine Oxidation Reactions. Redox Biol..

[20] Okoye C.N., Koren S.A., Wojtovich A.P. (2023). Mitochondrial Complex I ROS Production and Redox Signaling in Hypoxia. Redox Biol..

[21] Mercola J. (2025). Reductive Stress and Mitochondrial Dysfunction: The Hidden Link in Chronic Disease. Free Radic. Biol. Med..

[22] Auten R.L., Davis J.M. (2009). Oxygen Toxicity and Reactive Oxygen Species: The Devil Is in the Details. Pediatr. Res..

[23] De Almeida A.J.P.O., de Oliveira J.C.P.L., da Silva Pontes L.V., de Souza Júnior J.F., Gonçalves T.A.F., Dantas S.H., de Almeida Feitosa M.S., Silva A.O., de Medeiros I.A. (2022). ROS: Basic Concepts, Sources, Cellular Signaling, and Its Implications in Aging Pathways. Oxid. Med. Cell Longev..

[24] Adams R.A., Liu Z., Hsieh C., Marko M., Lederer W.J., Jafri M.S., Mannella C. (2023). Structural Analysis of Mitochondria in Cardiomyocytes: Insights into Bioenergetics and Membrane Remodeling. Curr. Issues Mol. Biol..

[25] Zorova L.D., Popkov V.A., Plotnikov E.Y., Silachev D.N., Pevzner I.B., Jankauskas S.S., Babenko V.A., Zorov S.D., Balakireva A.V., Juhaszova M. (2018). Mitochondrial Membrane Potential. Anal. Biochem..

[26] Bernardi P., Gerle C., Halestrap A.P., Jonas E.A., Karch J., Mnatsakanyan N., Pavlov E., Sheu S.-S., Soukas A.A. (2023). Identity, Structure, and Function of the Mitochondrial Permeability Transition Pore: Controversies, Consensus, Recent Advances, and Future Directions. Cell Death Differ..

[27] Kühlbrandt W. (2015). Structure and Function of Mitochondrial Membrane Protein Complexes. BMC Biol..

[28] Wollweber F., von der Malsburg K., van der Laan M. (2017). Mitochondrial Contact Site and Cristae Organizing System: A Central Player in Membrane Shaping and Crosstalk. Biochim. Biophys. Acta (BBA)-Mol. Cell Res..

[29] Kondadi A.K., Anand R., Reichert A.S. (2020). Cristae Membrane Dynamics—A Paradigm Change. Trends Cell Biol..

[30] Ryu K.W., Fung T.S., Baker D.C., Saoi M., Park J., Febres-Aldana C.A., Aly R.G., Cui R., Sharma A., Fu Y. (2024). Cellular ATP Demand Creates Metabolically Distinct Subpopulations of Mitochondria. Nature.

[31] Song Z., Ghochani M., McCaffery J.M., Frey T.G., Chan D.C. (2009). Mitofusins and OPA1 Mediate Sequential Steps in Mitochondrial Membrane Fusion. Mol. Biol. Cell.

[32] Gao S., Hu J. (2021). Mitochondrial Fusion: The Machineries In and Out. Trends Cell Biol..

[33] Tilokani L., Nagashima S., Paupe V., Prudent J. (2018). Mitochondrial Dynamics: Overview of Molecular Mechanisms. Essays Biochem..

[34] Han S., Zhao F., Hsia J., Ma X., Liu Y., Torres S., Fujioka H., Zhu X. (2021). The Role of Mfn2 in the Structure and Function of Endoplasmic Reticulum-Mitochondrial Tethering in Vivo. J. Cell Sci..

[35] Yao C.-H., Wang R., Wang Y., Kung C.-P., Weber J.D., Patti G.J. (2019). Mitochondrial Fusion Supports Increased Oxidative Phosphorylation during Cell Proliferation. eLife.

[36] Pyakurel A., Savoia C., Hess D., Scorrano L. (2015). Extracellular Regulated Kinase Phosphorylates Mitofusin 1 to Control Mitochondrial Morphology and Apoptosis. Mol. Cell.

[37] Ong S.-B., Kalkhoran S.B., Hernández-Reséndiz S., Samangouei P., Ong S.-G., Hausenloy D.J. (2017). Mitochondrial-Shaping Proteins in Cardiac Health and Disease—The Long and the Short of It!. Cardiovasc. Drugs Ther..

[38] Zerihun M., Sukumaran S., Qvit N. (2023). The Drp1-Mediated Mitochondrial Fission Protein Interactome as an Emerging Core Player in Mitochondrial Dynamics and Cardiovascular Disease Therapy. Int. J. Mol. Sci..

[39] Coronado M., Fajardo G., Nguyen K., Zhao M., Kooiker K., Jung G., Hu D.-Q., Reddy S., Sandoval E., Stotland A. (2018). Physiological Mitochondrial Fragmentation Is a Normal Cardiac Adaptation to Increased Energy Demand. Circ. Res..

[40] Rios L., Pokhrel S., Li S.-J., Heo G., Haileselassie B., Mochly-Rosen D. (2023). Targeting an Allosteric Site in Dynamin-Related Protein 1 to Inhibit Fis1-Mediated Mitochondrial Dysfunction. Nat. Commun..

[41] Ventura-Clapier R., Garnier A., Veksler V. (2008). Transcriptional Control of Mitochondrial Biogenesis: The Central Role of PGC-1α. Cardiovasc. Res..

[42] Liu L., Li Y., Chen G., Chen Q. (2023). Crosstalk between Mitochondrial Biogenesis and Mitophagy to Maintain Mitochondrial Homeostasis. J. Biomed. Sci..

[43] Tang Y., Mi C., Liu J., Gao F., Long J. (2014). Compromised Mitochondrial Remodeling in Compensatory Hypertrophied Myocardium of Spontaneously Hypertensive Rat. Cardiovasc. Pathol..

[44] Whitehead N., Gill J.F., Brink M., Handschin C. (2018). Moderate Modulation of Cardiac PGC-1α Expression Partially Affects Age-Associated Transcriptional Remodeling of the Heart. Front. Physiol..

[45] Narendra D.P., Jin S.M., Tanaka A., Suen D.-F., Gautier C.A., Shen J., Cookson M.R., Youle R.J. (2010). PINK1 Is Selectively Stabilized on Impaired Mitochondria to Activate Parkin. PLoS Biol..

[46] Vives-Bauza C., Zhou C., Huang Y., Cui M., de Vries R.L.A., Kim J., May J., Tocilescu M.A., Liu W., Ko H.S. (2010). PINK1-Dependent Recruitment of Parkin to Mitochondria in Mitophagy. Proc. Natl. Acad. Sci. USA.

[47] Geisler S., Holmström K.M., Skujat D., Fiesel F.C., Rothfuss O.C., Kahle P.J., Springer W. (2010). PINK1/Parkin-Mediated Mitophagy Is Dependent on VDAC1 and P62/SQSTM1. Nat. Cell Biol..

[48] Matsuda N., Sato S., Shiba K., Okatsu K., Saisho K., Gautier C.A., Sou Y.-S., Saiki S., Kawajiri S., Sato F. (2010). PINK1 Stabilized by Mitochondrial Depolarization Recruits Parkin to Damaged Mitochondria and Activates Latent Parkin for Mitophagy. J. Cell Biol..

[49] Wang S., Long H., Hou L., Feng B., Ma Z., Wu Y., Zeng Y., Cai J., Zhang D., Zhao G. (2023). The Mitophagy Pathway and Its Implications in Human Diseases. Signal Transduct. Target. Ther..

[50] Zhang H., Xie S., Deng W. (2024). Mitophagy in Doxorubicin-Induced Cardiotoxicity: Insights into Molecular Biology and Novel Therapeutic Strategies. Biomolecules.

[51] Doblado L., Lueck C., Rey C., Samhan-Arias A.K., Prieto I., Stacchiotti A., Monsalve M. (2021). Mitophagy in Human Diseases. Int. J. Mol. Sci..

[52] Shires S.E., Gustafsson Å.B. (2015). Mitophagy and Heart Failure. J. Mol. Med..

[53] Liu Y., Wang Y., Bi Y., Zhao Z., Wang S., Lin S., Yang Z., Wang X., Mao J. (2023). Emerging Role of Mitophagy in Heart Failure: From Molecular Mechanism to Targeted Therapy. Cell Cycle.

[54] Ai L., de Freitas Germano J., Huang C., Aniag M., Sawaged S., Sin J., Thakur R., Rai D., Rainville C., Sterner D.E. (2025). Enhanced Parkin-Mediated Mitophagy Mitigates Adverse Left Ventricular Remodelling after Myocardial Infarction: Role of PR-364. Eur. Heart J..

[55] Ma Y., Zhou X., Gui M., Yao L., Li J., Chen X., Wang M., Lu B., Fu D. (2024). Mitophagy in Hypertension-Mediated Organ Damage. Front. Cardiovasc. Med..

[56] Shires S., Gustafsson Å.B. (2018). Regulating Renewable Energy: Connecting AMPKα2 to PINK1/Parkin-Mediated Mitophagy in the Heart. Circ. Res..

[57] Chen W., Zhao H., Li Y. (2023). Mitochondrial Dynamics in Health and Disease: Mechanisms and Potential Targets. Signal Transduct. Target. Ther..

[58] Daghistani H.M., Rajab B.S., Kitmitto A. (2019). Three-dimensional Electron Microscopy Techniques for Unravelling Mitochondrial Dysfunction in Heart Failure and Identification of New Pharmacological Targets. Br. J. Pharmacol..

[59] Yoshii A., McMillen T.S., Wang Y., Zhou B., Chen H., Banerjee D., Herrero M., Wang P., Muraoka N., Wang W. (2024). Blunted Cardiac Mitophagy in Response to Metabolic Stress Contributes to HFpEF. Circ. Res..

[60] Frey T.G., Mannella C.A. (2000). The Internal Structure of Mitochondria. Trends Biochem. Sci..

[61] Brand M.D., Nicholls D.G. (2011). Assessing Mitochondrial Dysfunction in Cells. Biochem. J..

[62] Wu M., Neilson A., Swift A.L., Moran R., Tamagnine J., Parslow D., Armistead S., Lemire K., Orrell J., Teich J. (2007). Multiparameter Metabolic Analysis Reveals a Close Link between Attenuated Mitochondrial Bioenergetic Function and Enhanced Glycolysis Dependency in Human Tumor Cells. Am. J. Physiol.-Cell Physiol..

[63] Yang D., Liu H.-Q., Liu F.-Y., Guo Z., An P., Wang M.-Y., Yang Z., Fan D., Tang Q.-Z. (2022). Mitochondria in Pathological Cardiac Hypertrophy Research and Therapy. Front. Cardiovasc. Med..

[64] Chan S.H.H., Wu K.L.H., Chang A.Y.W., Tai M.-H., Chan J.Y.H. (2009). Oxidative Impairment of Mitochondrial Electron Transport Chain Complexes in Rostral Ventrolateral Medulla Contributes to Neurogenic Hypertension. Hypertension.

[65] Xue R.-Q., Zhao M., Wu Q., Yang S., Cui Y.-L., Yu X.-J., Liu J., Zang W.-J. (2019). Regulation of Mitochondrial Cristae Remodelling by Acetylcholine Alleviates Palmitate-Induced Cardiomyocyte Hypertrophy. Free Radic. Biol. Med..

[66] Lopaschuk G.D., Karwi Q.G., Tian R., Wende A.R., Abel E.D. (2021). Cardiac Energy Metabolism in Heart Failure. Circ. Res..

[67] Ritterhoff J., Tian R. (2017). Metabolism in Cardiomyopathy: Every Substrate Matters. Cardiovasc. Res..

[68] Liu M., Lv J., Pan Z., Wang D., Zhao L., Guo X. (2022). Mitochondrial Dysfunction in Heart Failure and Its Therapeutic Implications. Front. Cardiovasc. Med..

[69] Nguyen B.Y., Ruiz-Velasco A., Bui T., Collins L., Wang X., Liu W. (2019). Mitochondrial Function in the Heart: The Insight into Mechanisms and Therapeutic Potentials. Br. J. Pharmacol..

[70] Paraskevaidis I., Kourek C., Farmakis D., Tsougos E. (2024). Heart Failure: A Deficiency of Energy—A Path Yet to Discover and Walk. Biomedicines.

[71] Kuroda J., Sadoshima J. (2010). NADPH Oxidase and Cardiac Failure. J. Cardiovasc. Transl. Res..

[72] Zorov D.B., Juhaszova M., Sollott S.J. (2014). Mitochondrial Reactive Oxygen Species (ROS) and ROS-Induced ROS Release. Physiol. Rev..

[73] Peoples J.N., Saraf A., Ghazal N., Pham T.T., Kwong J.Q. (2019). Mitochondrial Dysfunction and Oxidative Stress in Heart Disease. Exp. Mol. Med..

[74] Dikalov S.I., Nazarewicz R.R. (2013). Angiotensin II-Induced Production of Mitochondrial Reactive Oxygen Species: Potential Mechanisms and Relevance for Cardiovascular Disease. Antioxid. Redox Signal.

[75] Bhullar S.K., Dhalla N.S. (2022). Angiotensin II-Induced Signal Transduction Mechanisms for Cardiac Hypertrophy. Cells.

[76] Ježek J., Cooper K.F., Strich R. (2021). The Impact of Mitochondrial Fission-Stimulated ROS Production on Pro-Apoptotic Chemotherapy. Biology.

[77] Giorgi C., Marchi S., Simoes I.C.M., Ren Z., Morciano G., Perrone M., Patalas-Krawczyk P., Borchard S., Jȩdrak P., Pierzynowska K. (2018). Mitochondria and Reactive Oxygen Species in Aging and Age-Related Diseases. Int. Rev. Cell Mol. Biol..

[78] Berk B.C., Fujiwara K., Lehoux S. (2007). ECM Remodeling in Hypertensive Heart Disease. J. Clin. Investig..

[79] Bosonea A.-M., Wang X., Odenbach J., Fernandez-Patron C. (2011). Metalloproteinases in Hypertension and Cardiac Disease: Differential Expression and Mutual Regulation. Drug Discov. Today Dis. Models.

[80] Ammarguellat F.Z., Gannon P.O., Amiri F., Schiffrin E.L. (2002). Fibrosis, Matrix Metalloproteinases, and Inflammation in the Heart of DOCA-Salt Hypertensive Rats: Role of ETA Receptors. Hypertension.

[81] Qi J., Wang F., Yang P., Wang X., Xu R., Chen J., Yuan Y., Lu Z., Duan J. (2018). Mitochondrial Fission Is Required for Angiotensin II-Induced Cardiomyocyte Apoptosis Mediated by a Sirt1-P53 Signaling Pathway. Front. Pharmacol..

[82] Zanfardino P., Amati A., Perrone M., Petruzzella V. (2025). The Balance of MFN2 and OPA1 in Mitochondrial Dynamics, Cellular Homeostasis, and Disease. Biomolecules.

[83] Nan J., Zhu W., Rahman M.S., Liu M., Li D., Su S., Zhang N., Hu X., Yu H., Gupta M.P. (2017). Molecular Regulation of Mitochondrial Dynamics in Cardiac Disease. Biochim. Biophys. Acta (BBA)-Mol. Cell Res..

[84] Zhang X., Li Z.-L., Crane J.A., Jordan K.L., Pawar A.S., Textor S.C., Lerman A., Lerman L.O. (2014). Valsartan Regulates Myocardial Autophagy and Mitochondrial Turnover in Experimental Hypertension. Hypertension.

[85] Eisenberg T., Abdellatif M., Schroeder S., Primessnig U., Stekovic S., Pendl T., Harger A., Schipke J., Zimmermann A., Schmidt A. (2016). Cardioprotection and Lifespan Extension by the Natural Polyamine Spermidine. Nat. Med..

[86] Chaanine A.H., Gordon R.E., Kohlbrenner E., Benard L., Jeong D., Hajjar R.J. (2013). Potential Role of BNIP3 in Cardiac Remodeling, Myocardial Stiffness and Endoplasmic Reticulum-Mitochondrial Calcium Homeostasis in Diastolic and Systolic Heart Failure. Circ. Heart Fail..

[87] Shao R., Li J., Qu T., Liao Y., Chen M. (2022). Mitophagy: A Potential Target for Pressure Overload-Induced Cardiac Remodelling. Oxid. Med. Cell Longev..

[88] Dridi H., Santulli G., Bahlouli L., Miotto M.C., Weninger G., Marks A.R. (2023). Mitochondrial Calcium Overload Plays a Causal Role in Oxidative Stress in the Failing Heart. Biomolecules.

[89] Krstic A.M., Power A.S., Ward M.-L. (2023). Increased Mitochondrial Calcium Fluxes in Hypertrophic Right Ventricular Cardiomyocytes from a Rat Model of Pulmonary Artery Hypertension. Life.

[90] Hurst S., Hoek J., Sheu S.-S. (2017). Mitochondrial Ca^2+^ and Regulation of the Permeability Transition Pore. J. Bioenerg. Biomembr..

[91] Weiss J.N., Korge P., Honda H.M., Ping P. (2003). Role of the Mitochondrial Permeability Transition in Myocardial Disease. Circ. Res..

[92] Murphy E., Ardehali H., Balaban R.S., DiLisa F., Dorn G.W., Kitsis R.N., Otsu K., Ping P., Rizzuto R., Sack M.N. (2016). AHA Position Paper on Mitochondrial Function, Biology and Role in Disease. Circ. Res..

[93] Kiyuna L.A., e Albuquerque R.P., Chen C.-H., Mochly-Rosen D., Ferreira J.C.B. (2018). Targeting Mitochondrial Dysfunction and Oxidative Stress in Heart Failure: Challenges and Opportunities. Free Radic. Biol. Med..

[94] Strauss M.H., Hall A.S., Narkiewicz K. (2023). The Combination of Beta-Blockers and ACE Inhibitors Across the Spectrum of Cardiovascular Diseases. Cardiovasc. Drugs Ther..

[95] Yang M.-Q., Zhang S.-L., Sun L., Huang L.-T., Yu J., Zhang J.-H., Tian Y., Han C.-B., Ma J.-T. (2024). Targeting Mitochondria: Restoring the Antitumor Efficacy of Exhausted T Cells. Mol. Cancer.

[96] Madreiter-Sokolowski C.T., Hiden U., Krstic J., Panzitt K., Wagner M., Enzinger C., Khalil M., Abdellatif M., Malle E., Madl T. (2024). Targeting Organ-Specific Mitochondrial Dysfunction to Improve Biological Aging. Pharmacol. Ther..

[97] Sharma A., Smith H.J., Yao P., Mair W.B. (2019). Causal Roles of Mitochondrial Dynamics in Longevity and Healthy Aging. EMBO Rep..

[98] Srivastava S. (2017). The Mitochondrial Basis of Aging and Age-Related Disorders. Genes.

[99] Wang Y., Li Y., He C., Gou B., Song M. (2019). Mitochondrial Regulation of Cardiac Aging. Biochim. Biophys. Acta (BBA)-Mol. Basis Dis..

[100] Sprason C., Tucker T., Clancy D. (2024). MtDNA Deletions and Aging. Front. Aging.

[101] Judge S., Jang Y.M., Smith A., Hagen T., Leeuwenburgh C. (2005). Age-Associated Increases in Oxidative Stress and Antioxidant Enzyme Activities in Cardiac Interfibrillar Mitochondria: Implications for the Mitochondrial Theory of Aging. FASEB J..

[102] Chen S., Li Q., Shi H., Li F., Duan Y., Guo Q. (2024). New Insights into the Role of Mitochondrial Dynamics in Oxidative Stress-Induced Diseases. Biomed. Pharmacother..

[103] Owada T., Yamauchi H., Saitoh S.-I., Miura S., Machii H., Takeishi Y. (2017). Resolution of Mitochondrial Oxidant Stress Improves Aged-Cardiovascular Performance. Coron. Artery Dis..

[104] Chiao Y.A., Zhang H., Sweetwyne M., Whitson J., Ting Y.S., Basisty N., Pino L.K., Quarles E., Nguyen N.-H., Campbell M.D. (2020). Late-Life Restoration of Mitochondrial Function Reverses Cardiac Dysfunction in Old Mice. eLife.

[105] Sagar S., Gustafsson A.B. (2023). Cardiovascular Aging: The Mitochondrial Influence. J. Cardiovasc. Aging.

[106] Vega R.B., Kelly D.P. (2017). Cardiac Nuclear Receptors: Architects of Mitochondrial Structure and Function. J. Clin. Investig..

[107] Birker K., Ge S., Kirkland N.J., Theis J.L., Marchant J., Fogarty Z.C., Missinato M.A., Kalvakuri S., Grossfeld P., Engler A.J. (2023). Mitochondrial MICOS Complex Genes, Implicated in Hypoplastic Left Heart Syndrome, Maintain Cardiac Contractility and Actomyosin Integrity. eLife.

[108] Jenkins B.C., Neikirk K., Katti P., Claypool S.M., Kirabo A., McReynolds M.R., Hinton A. (2024). Mitochondria in Disease: Changes in Shapes and Dynamics. Trends Biochem. Sci..

[109] Dungan C.M., Murach K.A., Frick K.K., Jones S.R., Crow S.E., Englund D.A., Vechetti I.J., Figueiredo V.C., Levitan B.M., Satin J. (2019). Elevated myonuclear density during skeletal muscle hypertrophy in response to training is reversed during detraining. Am. J. Physiol. Cell Physiol..

[110] Mitchell W., Pharaoh G., Tyshkovskiy A., Campbell M., Marcinek D.J., Gladyshev V.N. (2025). The Mitochondria-Targeted Peptide Therapeutic Elamipretide Improves Cardiac and Skeletal Muscle Function During Aging Without Detectable Changes in Tissue Epigenetic or Transcriptomic Age. Aging Cell.

[111] Verma S., Rawat S., Ho K.L., Wagg C.S., Zhang L., Teoh H., Dyck J.E., Uddin G.M., Oudit G.Y., Mayoux E. (2018). Empagliflozin Increases Cardiac Energy Production in Diabetes. JACC Basic. Transl. Sci..

[112] Zannad F., Ferreira J.P., Pocock S.J., Anker S.D., Butler J., Filippatos G., Brueckmann M., Ofstad A.P., Pfarr E., Jamal W. (2020). SGLT2 Inhibitors in Patients with Heart Failure with Reduced Ejection Fraction: A Meta-Analysis of the EMPEROR-Reduced and DAPA-HF Trials. Lancet.

[113] Deng Y., Li S., Chen Z., Wang W., Geng B., Cai J. (2021). Mdivi-1, a Mitochondrial Fission Inhibitor, Reduces Angiotensin-II- Induced Hypertension by Mediating VSMC Phenotypic Switch. Biomed. Pharmacother..

[114] De Wit-Verheggen V.H.W., Schrauwen-Hinderling V.B., Brouwers K., Jörgensen J.A., Schaart G., Gemmink A., Nascimento E.B.M., Hesselink M.K.C., Wildberger J.E., Segers P. (2023). PCr/ATP Ratios and Mitochondrial Function in the Heart. A Comparative Study in Humans. Sci. Rep..

